# Aluminum-Induced Alterations to the Cell Wall and Antioxidant Enzymes Involved in the Regulation of the Aluminum Tolerance of Chinese Fir (*Cunninghamia lanceolata*)

**DOI:** 10.3389/fpls.2022.891117

**Published:** 2022-04-28

**Authors:** Shanshan Xu, Lihua Wu, Lingyan Li, Minghui Zhong, Ying Tang, Guangqiu Cao, Kaimin Lin, Yiquan Ye

**Affiliations:** ^1^College of Forestry, Fujian Agriculture and Forestry University, Fuzhou, China; ^2^Chinese Fir Engineering Technology Research Center of National Forestry and Grassland Administration, Fuzhou, China

**Keywords:** aluminum, Chinese fir, cell wall, pectin methylation, pectin, hemicellulose

## Abstract

Chinese fir (*Cunninghamia lanceolata*), which is an important coniferous tree species in China, is mainly planted in acidic soils with toxic aluminum (Al) levels. However, the consequences of Al toxicity and its resistance mechanism in Chinese fir remain largely uncharacterized. In this study, the Al-induced modification and possible role of cell wall in regulating Al tolerance in Chinese fir were investigated by using seedlings with contrasting Al tolerance, namely, Al-sensitive (YX02) and Al-resistant (YX01) genotypes. The results in present work showed that Al treatment resulted in a dose- and time-dependent inhibition of root growth and oxidative damage in both genotypes, but more in YX02 than in YX01. The severe oxidative damage observed in YX02 under Al stress was found to correlate with lower antioxidant enzyme activities as compared with YX01. The greater root growth inhibition observed in YX02 compared with YX01 was associated with a higher accumulation of Al in pectin and hemicllulose 1 (HC1) fraction because of the higher pectin and HC1 contents and the lower degree of pectin demethylation due to enhanced pectin methylesterase activity in YX02, which ultimately enhanced cell wall binding capacity for Al in YX02. Taken together, our results suggested that enhancement of antioxidant enzyme activities and cell wall modification-induced Al exclusion are the two mechanisms responsible for the Al tolerance of Chinese fir.

## Introduction

Acidic soils (pH < 5.5) are prevalent in many regions worldwide, with approximately 30% of the world’s ice-free land affected by excessive acidity (Brunner and Sperisen, 2013). Recently, the soil acidification problem has become more seriously due to misuse or excessive use of fertilizers as well as acid deposition (von-Uexkull and Mutert, 1995; Duan et al., 2016). Under acidic soil condition, plants may suffer from various toxicities, including aluminum (Al), iron (Fe), proton (H^+^) (Kochian et al., 2005; de Wit et al., 2010; Fang et al., 2016; Rao et al., 2016). Since Al ion (Al^3+^) is reactive, it can interact with multiple cell components (Singh et al., 2017). These interactions result in inhibited of cell division and expansion, decreased plasma membrane integrity, and disrupted signaling cascades, and consequent the root elongation and uptake of water and nutrient elements were inhibited (Matsumoto, 2000; Kochian et al., 2015), which ultimately lead to crop yield losses. Therefore, Al toxicity is recognized as the most serious constraining factors affecting crop production in acidic soils. To deal with Al toxicity, plants have evolved a number of strategies, which can be broadly ascribed to external and internal resistance mechanisms (Horst et al., 2010). The external mechanisms is achieved by secreting chelating agents, such as organic acids, P_*i*_, or phenolic compounds from the root apex and the formation of non-toxic Al-chelating complexes in the rhizosphere to exclude Al from the roots (Kochian et al., 2015). In contrast, the internal mechanisms mainly involve the chelation of Al with organic substances in cells and then sequestration them into vacuoles (Wang et al., 2010, 2017; Chen et al., 2012; Zhou et al., 2014), or an enhancement of the cellular antioxidant capacities (Sun et al., 2014, 2015).

In addition to the above-mentioned mechanisms, recent evidence demonstrated that Al-induced alterations in cell wall properties and the associated Al exclusion are also critical for regulating plant Al tolerance (Zheng, 2014). For instance, Zhu et al. (2019a) showed that exogenous application of putrescine can significantly alleviate Al toxicity in rice (*Oryza sativa*) by lowering the Al content in the root cell wall via decreased pectin content and pectin methylesterases (PME) activity. The results of another recent study indicated that boron can reduce the apoplastic Al accumulation in trifoliate orange by decreasing the un-methylated pectin and hemicellulose (HC) contents and increasing pectin methylation, thereby decreasing the Al content in roots to prevent Al toxicity (Riaz et al., 2019). Moreover, a comparison of pea (*Pisum sativum* L.) cultivars with contrasting susceptibilities to Al toxicity revealed that the pectin content and PME activity are often greater in roots of Al-sensitive plants as compared with Al-resistant ones under Al stress (Li et al., 2016). Similar results also observed in buckwheat (*Fagopyrum tataricum*), it has been found that the higher retention of Al in roots of Al-sensitive cultivar “Liuku2” always accompanied with higher low-methyl-ester pectin content and PME activity (Yang et al., 2011a). In addition to pectin, there is accumulating evidence showed that the cell wall HC is also vital for regulating plant Al tolerance (Yang et al., 2011b; Zhu et al., 2017; Li et al., 2020). More specifically, Yang et al. (2008) found a negative correlation between HC content and Al accumulation in rice roots, the lower HC content could lead to a greater Al exclusion from roots in Al-resistant rice cultivar. Similarly, defects in the synthesis of xyloglucan, which is major component of HC, in the Arabidopsis mutant *xth31* considerably enhances plant Al-tolerance by decreasing the HC content and inhibiting the accumulation of Al in the roots (Zhu et al., 2012b). The results of another study suggest that the *O*-acetylation of the xyloglucan in HC may affect the Al-binding capacity of HC, which affects Al sensitivity (Zhu et al., 2014). Therefore, these results imply that decreasing the cell wall Al content by modifying cell wall components and properties is also a major mechanism employed by plants under Al stress.

Presently, most of the studies related to Al toxicity in plants have focused on herbaceous species, with relatively few studies investigated the role of the cell wall in regulating Al toxicity in woody plant species, especially in coniferous trees (Ryder et al., 2003; Prabagar et al., 2011). Different plant species as well as diverse genotypes within the same species may respond differently to Al stress. Therefore, whether Al-induced cell wall modifications are critical for regulating the Al tolerance of coniferous trees remains far from clear. Chinese fir is an important coniferous tree species in southern China (Ma et al., 2007). Because of its substantial economical value, Chinese fir is now the primary tree species used for afforestation in China, wherein it is grown on more than over 24% of the artificial forest area (Zhang et al., 2004). However, most of the Chinese fir trees were planted in iron-rich aluminized acidic soils, making Al toxicity a major factor limiting tree growth and yield (Luo et al., 2018a,b). Unfortunately, the effects of Al toxicity and the associated defense responses of Chinese fir remain largely unknown. In this study, the Al-induced immobilization of Al in the cell wall and its underlying mechanisms were investigated in the root tips of two Chinese fir genotypes differentially tolerant to Al stress. Evidence presented in this work suggest that enhancing of antioxidant enzyme activities and the cell wall modification-induced Al exclusion from roots under Al stress are the two mechanisms employed by Chinese fir to increase its Al tolerance. To our knowledge, this is the first report to reveal the contribution of the cell wall to the Al resistance mechanism of Chinese fir.

## Materials and Methods

### Plant Materials and Growth Conditions

This study was using two Chinese fir genotypes that differ regarding their Al tolerance [YX01 (Al-resistant) and YX02 (Al-sensitive)]. The seeds of Chinese fir were provided by the Chinese Fir Engineering Technology Research Center of National Forestry and Grassland Administration. The seeds were first rinsed with water (three times) to eliminate the empty and aborted seeds and other particles. the remaining seeds were surface-sterilized with a 0.3% potassium permanganate solution for 30 min and then washed with ultrapure water for six times. The sterilized seeds were soaked in warm water (50°C) and allowed to cool down to room temperature (25°C) for 24 h. The soaked seeds were germinated and the resulting seedlings were grown in 3 mM CaCl_2_ solution on cardboard filter paper for 30 days. Uniformly growing seedlings were selected and pre-cultured in full-strength nutrient solution (SNS) (pH adjusted to 5.5) in a culture incubator for another 30 days. The composition of the nutrient solution was described in our previous report (Ye et al., 2019). The seedlings were placed in a controlled environment growth room as previously described (Ye et al., 2019). After pre-culturing, the uniformly growing Chinese fir seedlings were transferred to different treatment solutions: 1/2 SNS (CK) or 1/2 SNS supplemented with 1 mM AlCl_3_ (Al treatment) and treated for 16 days. During the treatment period, the solutions pH was adjusted to 4.5. Additionally, the solutions were aerated with an air pump and refreshed every 2 days.

### Root Elongation Determination

Two-month-old Chinese fir seedlings were cultured in the control (1/2 SNS, pH = 4.5) or Al treatment (1/2 SNS with 1 mM AlCl_3_, pH = 4.5) solutions for 16 days. At the start and end of the treatment period, the lengths of the primary roots of Chinese fir seedlings were measured with a ruler. The relative root elongation (%) of the seedlings was calculated by comparing the root elongation in the Al treatment solution with that in control solution.

### Membrane Lipid Peroxidation Determination

Membrane lipid peroxidation was determined as described by Zhu et al. (2012a). Briefly, the content of malondialdehyde (MDA) was determined by thiobarbituric acid reaction.

### Measurement of the Hydrogen Peroxide Content

The treated root tips (0-1 cm) of Chinese fir were harvested, after which the H_2_O_2_ content was determined according to the colorimetrical method as described by Jin et al. (2013). Briefly, the root tips of Chinese fir were ground in a mortar containing with ice-cold acetone and quartz sand. The ground material was centrifuged (12000 rpm for 10 min at 4°C) and the pelleted residue was discarded. A 1-ml aliquot of the supernatant was transferred into a 10-ml test tube, to which 1 ml 5% titanium sulfate solution and 2 ml 17 M ammonia solution were added into the test tube for forming precipitated. The precipitated material was collected and rinsed with ice-cold acetone for 3 times, and then dissolved in 5 ml 2 M H_2_SO_4_. The absorbance of the solution as well as a blank control solution was measured at 410 nm, after which the hydrogen peroxide content was calculated by the difference in the absorbance of the two solutions.

### Measurement of Root Activity

The root activity of Chinese fir was analyzed using triphenyl tetrazolium chloride (TTC) method. In brief, after treatment the roots of Chinese fir seedlings were rinsed with deionized water, then root tips (0.2 g) were collected and placed in 50-ml centrifuge tubes, 5 ml 0.4% TTC reagent and 5 ml 67 mM phosphatic buffer solution (pH = 7.0) were added to the tubes, which were then incubated at 37°C for 2 h in the darkness. The reaction was stopped by adding 2 ml of H_2_SO_4_ (1 M) into the tubes. After that the roots were transferred into mortar ground into homogenized and extracted with 5 ml ethyl acetate, the residue were rinsed with ethyl acetate for three times, then the red supernatant were pooled into 20 ml volumetric flask. Solution absorbance was detected at 485 nm against blanks. The blank solution was prepared using the same reagents, but order in which the reagents were added differed (i.e., H_2_SO_4_ added first and then the roots and the other reagents).

### Cell Wall Fraction and Cell Wall Components Measurement

The preparation of cell wall was according to Ye et al. (2015). In brief, after treatment, the roots (0-10 mm) of Chinese fir was collected and rinsed with ultrapure water before they were ground in liquid nitrogen. Next, 10 mL of ethanol (75%) was added to the ground material, which was then incubated for 20 min. The sample was centrifuged at 10,000 × g for 20 min. The pelleted material was rinsed with acetone (for 15 min), then with methanol: chloroform = 1:1, v/v (for 15 min), at last rinsed with methanol (for 15 min). The final residues comprising the cell wall was collected, lyophilized, and stored in a refrigerator until analyzed. The pectin, hemicellulose 1 (HC1), and hemicellulose 2 (HC2) were fractionated as described in Yang et al. (2011b). The uronic acid content in the pectin, HC1, and HC2 was assayed by using a known galacturonic acid (GalA) calibration standard curve as described in previous (Blumenkrantz and Asboe-Hansen, 1973).

### Determination of the Aluminum Content

The Al contents in the root tips as well as in the cell wall and its fractions were analyzed as described by Yang et al. (2011b) and Zhu et al. (2017). Briefly, the Al contents in root tips (0–1 cm), cell wall and its fraction were extracted with 2 M HCl for 24 h. After extraction, the extract solutions were centrifuged at 15,000 × g for 10 min. The Al contents of the supernatants were determined by inductively coupled plasma optical emission spectrometry (ICP-OES).

### Analysis of the Activities of Antioxidant Enzyme

At the end of Al treatment, root tips of Chinese fir were collected and extracted with 2 mL buffer (50 mM PBS, pH 7.0, 1 mM EDTA and 1% PVP) after homogenized with liquid nitrogen. The extract was centrifuged at 12,000 × *g* for 10 min under 4°C, then the supernatant was used for enzyme activity analysis as described by Sun et al. (2014, 2015).

### Analysis of Pectin Methylesterase Activity

For pectin methylesterase (PME) activity analysis, the root tips (0-10 mm) of Chinese fir were extracted with buffer solution (100 mM Tris and 1M NaCl, pH 7.5) for 1 h at 4°C. After a centrifugation at 15,000 × *g* for 15 min at 4°C, the pelleted material was discarded and the PME activity of the supernatant was measured as described by Zhu et al. (2012a).

### Analysis of the Degree of Pectin Methylation

The degree of pectin methylation was determined by calculating the molar ratio of methanol content to uronic acid content as described in Ye et al. (2015). Methanol content released from pectin was measured according to Ye et al. (2015), namely, pectin methyl ester in pectin fraction was hydrolyzed by adding 10 mL of 1 M KOH into 5 mL pectin solutions in a centrifuge tube (50 mL), and incubated for 40 min (25°C). Then the pectin hydrolysates solutions were neutralized with H_3_PO_4_ to give 20 mL volume. Isopyknic alcohol oxidase (1 UN⋅mL^–1^) solution was then added into above solutions and kept at 25°C for 15 min. Finally, fluoral-P (2 mL) was added and incubated at 60°C for 15 min before cooled down to 25°C and determined at 412 nm by spectrophotometer. The uronic acid content in the pectin was determined as described above.

### Aluminum Adsorption Kinetics

Cell wall extraction was described as previous. Cell wall from CK and Al treatment were used for Al adsorption kinetics experiment. Before the experiment, the cell wall from Al-treated sample was reactivated by two 24-h treatments with 2 M HCl. After rinsing with deionized water, the reactivated cell wall was freeze-dried for further used. The cell wall Al adsorption kinetics experiment were carried out according to Zheng et al. (2004). In brief, the adsorption solution (0.5 mM CaCl_2_ and 20 μM AlCl_3_, pH 4.5) was run through a column filled with 20 mg of cell wall at a speed of 3 mL⋅15 min^–1^ by fraction collector, and then Al content in eluate was detected by ICP-OES.

### Statistical Analysis

Data were analyzed using the DPS software (version 9.5). Specifically, the data were subjected to a one-way analysis of variance and the means of each treatment were compared by the least significant difference test. Different letters on the histograms indicate statistically different at *P* < 0.05. * or ^**^ above the lines indicate statistically different at *P* < 0.05 and *P* < 0.01, respectively.

## Results

### Effects of the Aluminum Treatment on Root Elongation

**FIGURE 1:**
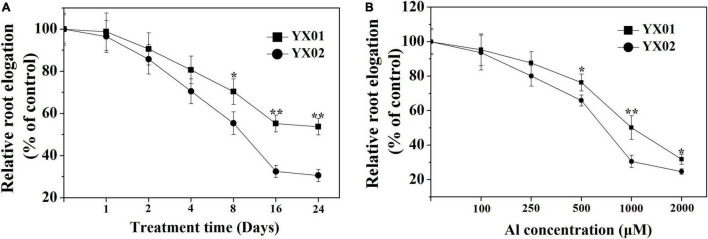
Effects of different aluminum (Al) exposure time **(A)** and concentration **(B)** on relative root elongation of Chinese fir seedlings. Two-month-old Chinese fir seedlings were exposed to 1/2 strength complete nutrition solution without Al (CK), or with 1 mM AlCl_3_ (Al) for 24 d, or exposed to CK or different concentration Al for 16 d. Bars are means of four replicates ± SD. * or ** above lines represent significant differences at *P < 0.05* and *P < 0.0*1, respectively.

Root growth inhibition is a typical symptom for plants under Al stress. Hence, root growth responses in Chinese fir were analyzed. Decreased root growth was observed for both genotypes as the Al treatment time increased (Figure 1A). When the exposure time exceeded 8 days, a significant difference in root elongation was detected between YX01 and YX02, and this difference became more evident as the exposure time increased to 16 days. However, Al exposure time longer 16 days did not lead to a further increase in the difference between YX01 and YX02. Similarly, the inhibitory effects of Al stress on root growth worsened as the Al concentration increased, with root growth inhibition reaching approximately 50% at 1000 μM Al (Figure 1B). Therefore, 1000 μM Al and 16 days of Al treatment period was employed in the following experiments.

### Effects of the Aluminum Treatment on Oxidative Damage, Root Activity, and the Root Aluminum Content

**FIGURE 2:**
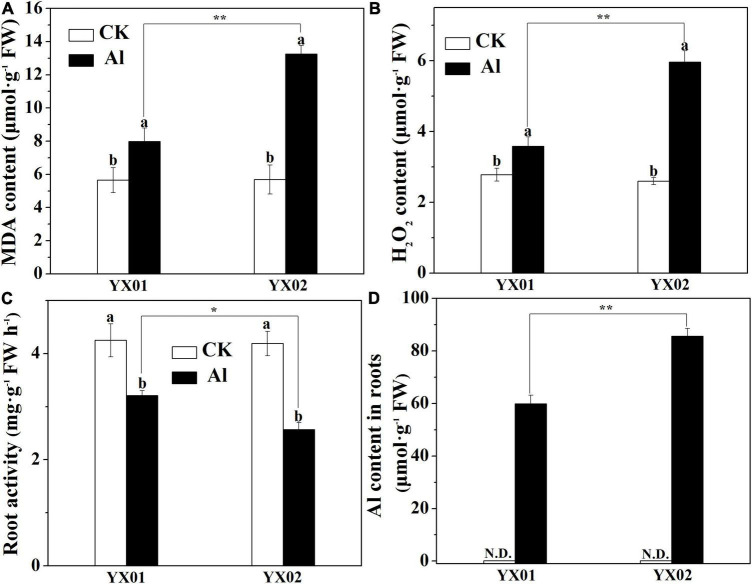
Effects of Al treatment on malondialdehyde (MDA) content **(A)**, hydrogen peroxide content **(B)**, root activity **(C)** and Al content **(D)** in roots of Chinese fir seedlings. Two-month-old Chinese fir seedlings were exposed to 1/2 strength complete nutrition solution without Al (CK), or with 1 mM AlCl_3_ (Al) for 16 d. After treatment, root tips of Chinese fir were collected for analysis. Bars are means of four replicates ± SD. Different letters above histogram represent significant differences at *P < 0.05*. * or ** above lines represent significant differences between genotypes at *P < 0.05* and *P < 0.0*1, respectively. N.D. indicates not detected.

The Al treatment resulted in significantly increased of MDA and H_2_O_2_ contents in root tips of Chinese fir as compared with control (Figures 2A,B). Additionally, a more significantly higher accumulation of MDA and H_2_O_2_ contents was observed in Al-sensitive genotype YX02 when compared with Al-resistant genotype YX01. Moreover, the exposure to Al stress significantly decreased the root activity, with a greater decrease in YX02 than in YX01 (Figure 2C). Similar to the MDA and H_2_O_2_ contents, Al exposure resulted in a significantly increased Al accumulation in the roots of both genotypes, with a more significantly higher of Al content found in the roots of YX02 (Figure 2D).

### Effects of Aluminum Stress on Antioxidant Enzyme Activities

**FIGURE 3:**
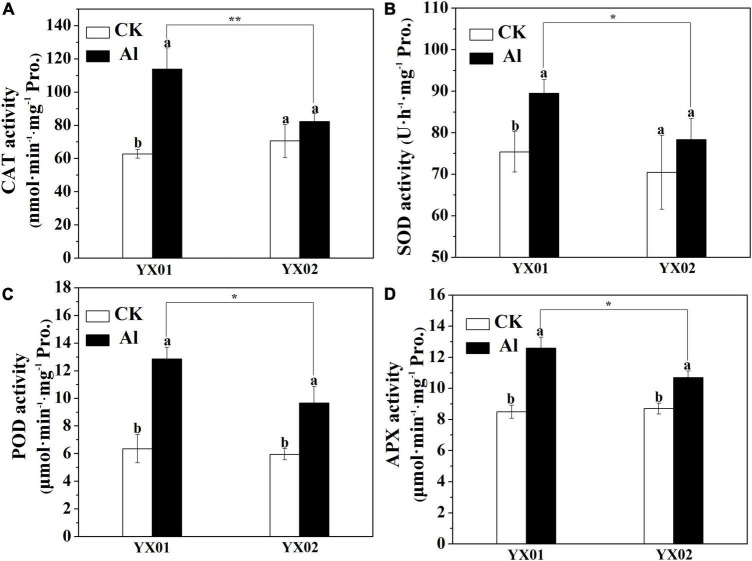
Effects of Al treatment on the activities catalase (CAT) enzyme **(A)**, superoxide dismutase (SOD) enzyme **(B)**, peroxidase (POD) enzyme **(C)**, and ascorbate peroxidase (APX) enzyme **(D)** in roots of Chinese fir seedlings. Two-month-old Chinese fir seedlings were exposed to 1/2 strength complete nutrition solution without Al (CK), or with 1 mM AlCl_3_ (Al) for 16 d. After treatment, root tips of Chinese fir were collected for analysis. Bars are means of four replicates ± SD. Different letters above histogram represent significant differences at *P < 0.05*. * or ** above lines represent significant differences between genotypes at *P < 0.05* and *P < 0.0*1, respectively.

Plants commonly respond to Al stress by enhancing antioxidant enzyme activities to increase the scavenging of reactive oxygen species (ROS), thereby minimizing the effects of Al toxicity (Sun et al., 2015). In the current study, the catalase (CAT), superoxide dismutase (SOD), peroxidase (POD), and ascorbate peroxidase (APX) activities significantly increased in both genotypes following the Al treatment (Figure 3). Furthermore, the CAT, SOD, POD, and APX activities were substantially higher in the YX01 roots than in the YX02 ones (Figure 3). These results indicated that Chinese fir can initiate antioxidant-related defenses under Al stress to eliminate ROS. Moreover, the capacity to scavenge ROS is greater in YX01 than in YX02.

### Effects of Aluminum Treatment on Aluminum Content in Cell Wall Polysaccharides

**FIGURE 4:**
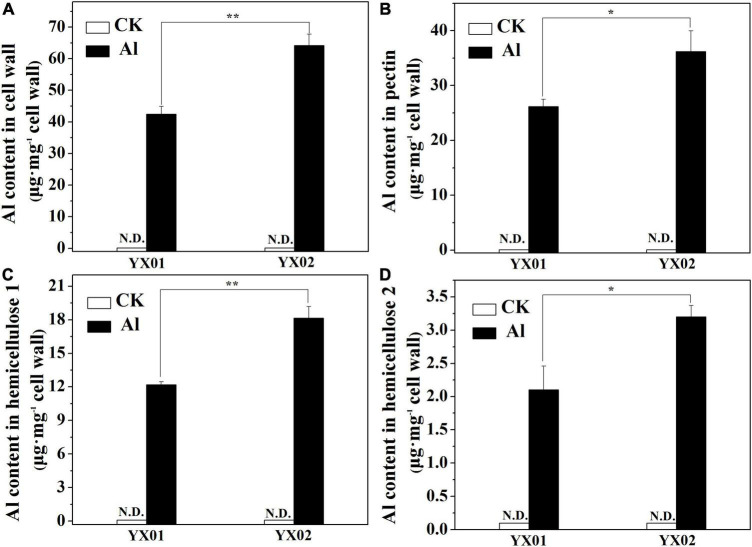
Effects of Al treatment on Al content in cell wall **(A)**, pectin **(B)**, hemicellulose 1 (HC1) **(C)** and hemicellulose 2 (HC2) **(D)** of Chinese fir seedlings. Two-month-old Chinese fir seedlings were exposed to 1/2 strength complete nutrition solution without Al (CK), or with 1 mM AlCl_3_ (Al) for 16 d. After treatment, cell wall was extracted from root tips of Chinese fir and fraction into pectin, HC1, and HC2 for Al content determination. Bars are means of four replicates ± SD. Different letters above histogram represent significant differences at *P < 0.05*. * or ** above lines represent significant differences between genotypes at *P < 0.05* and *P < 0.0*1, respectively. N.D. indicates not detected.

We further analyzed the Al content in the cell wall and its fractions because it is the major sites for Al accumulated in roots. Al was undetectable in the control. In contrast, the Al treatment significantly increased the cell wall Al content in both genotypes, but the Al content was significantly lower in YX01 than in YX02 (Figure 4A). A similar trend was observed when we analyzed the Al content in the cell wall polysaccharides. Consistent with the cell wall Al content, Al was not detected in the pectin, HC1, and HC2 of the control samples, whereas the Al treatment significantly increased the Al content in pectin, HC1 and HC2 (Figures 4B–D). More specifically, the Al content in pectin, HC1, and HC2 was, respectively, 38.12%, 49.09%, and 52.38% greater in YX02 than in YX01 (Figures 4B–D). We also found that Al was most abundant in pectin and HC1, implying these two cell wall components are the major Al-binding sites in Chinese fir roots.

### Effects of Aluminum Treatment on Cell Wall Polysaccharide Content

**FIGURE 5:**
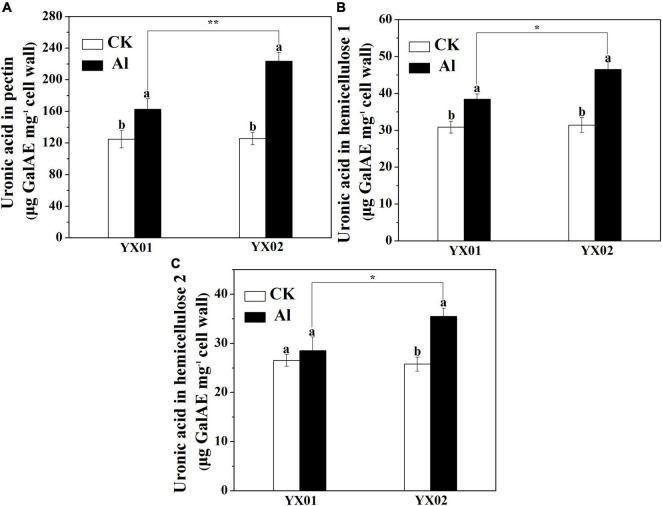
Effects of Al treatment on uronic acid content in root tips of Chinese fir seedlings. Two-month-old Chinese fir seedlings were exposed to 1/2 strength complete nutrition solution without Al (CK), or with 1 mM AlCl_3_ (Al) for 16 d. After treatment, cell wall was extracted from root tips of Chinese fir and fraction into pectin **(A)**, HC1 **(B)**, and HC2 **(C)** for uronic acid measurement. Bars are means of four replicates ± SD. Different letters above histogram represent significant differences at *P < 0.05*. * or ** above lines represent significant differences between genotypes at *P < 0.05* and *P < 0.0*1, respectively.

Previous research revealed that the content cell wall polysaccharide determines the binding capacities of cell wall for metal ions. Accordingly, we analyzed the cell wall polysaccharide content. A significantly increased in pectin and HC1 contents was observed in roots of Chinese fir exposed to Al as compared with control. Additionally, the contents of pectin and HC1 in the roots of YX02 were significantly higher than that of YX01 (Figures 5A,B). Compared with the control samples, the exposure to Al stress also increased the HC2 content in the roots of both genotypes, although not significantly for YX01, However, the HC2 content was significantly higher in YX02 than in YX01 (Figure 5C).

### Effects of the Aluminum Treatment on Pectin Methylesterase Activity and the Degree of Pectin Methyl-Esterification

**FIGURE 6:**
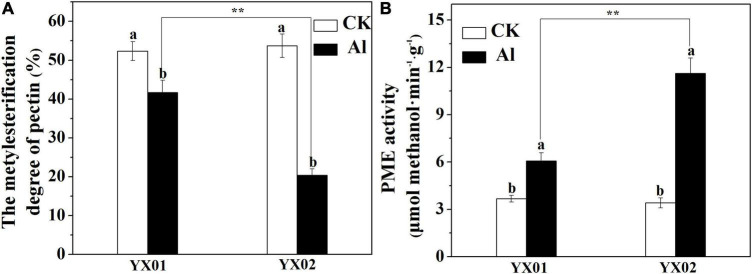
Effects of Al treatment on the degree of pectin methylesterification **(A)** and pectin methylesterase **(B)** in root tips of Chinese fir seedlings. Two-month-old Chinese fir seedlings were exposed to 1/2 strength complete nutrition solution without Al (CK), or with 1 mM AlCl_3_ (Al) for 16 d. After treatment, root tips of Chinese fir were collected for further analysis. Bars are means of four replicates ± SD. Different letters above histogram represent significant differences at *P < 0.05*. ** above lines represent significant differences between genotypes at *P < 0.0*1, respectively.

The degree of pectin methyl-esterification (DM), due to PME activity is another important factor influencing metal ions accumulated in the cell wall. As shown in Figure 6A, the Al treatment leads to a significant decrease in the DM, relative to the control, especially in the Al-sensitive genotype YX02. The DM was 51.22% lower in the YX02 roots than in the YX01 roots following the Al treatment. Consistent with the decreased DM, the PME activity significantly increased in both genotypes treated with Al, with a greater increase in the Al-sensitive genotype (Figure 6B).

### Different in the Aluminum-Binding Capacity of the Cell Wall of Chinese Fir Genotypes Under Aluminum Stress

**FIGURE 7:**
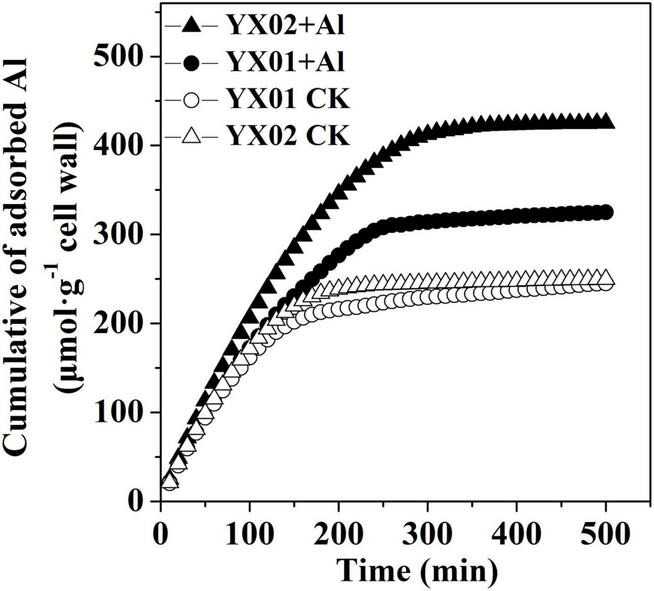
Al adsorption kinetic of cell wall from Chinese fir roots. Two-month-old Chinese fir seedlings were exposed to 1/2 strength complete nutrition solution without Al (CK), or with 1 mM AlCl_3_ (Al) for 16 d. After treatment, root tips of Chinese fir were collected for cell wall extraction and analysis.

Cell wall adsorption kinetics experiments were carried out by using cell wall from control and Al treatment of both genotypes to test whether the difference in cell wall Al accumulation both genotypes is associated with their difference in Al binding capacity. We found that the capacity of the cell wall to adsorb Al did not varied significantly between the two genotypes under control conditions. The Al treatment induced a considerable increase in the Al adsorption capacity of both genotypes. The cell wall of the Al-treated YX01 and YX02 roots adsorbed 324.85 μmol g^–1^ and 425.22 μmol g^–1^Al, respectively (Figure 7), suggesting Al-induced modifications in the cell wall enhanced the cell wall Al-binding capacity, resulting in enhanced Al accumulation in the cell wall.

## Discussion

Root growth inhibition is recognized as one of the most typical symptoms of Al toxicity, which may resulted from inhibition of cell division and expansion, disruption of plasma membrane integrity (Silva et al., 2012; Liu et al., 2018). Therefore, Al-induced root growth inhibition is frequently used as an indicator of the Al tolerance of many plants, including wheat (*Triticum aestivum* L.) (Sun et al., 2020), Arabidopsis (*Arabidopsis thaliana*) (Lou et al., 2019), rice (*Oryza sativa*) (Zhu et al., 2017, 2019b), pea (*Pisum sativum* L.) (Li et al., 2016), trifoliate orange [*Poncirus trifoliata* (L.) Raf.] (Riaz et al., 2019), lettuce (*Lactuca sativa*) (Chen et al., 2020), and even coniferous trees (Hirano et al., 2012). In the present study, therefore, the variations in root growth under Al stress between two Chinese fir genotypes with contrasting Al tolerances were compared. Consistent with previous findings, in our time- and concentration-dependent Al-exposure experiments, Al treatment significantly restricted root elongation in both genotypes (Figure 1). Moreover, the genotypes responded differently to Al stress in terms of relative root elongation, with root elongation inhibited significantly more in the Al-sensitive genotype YX02 (Figure 1). There is accumulating evidence that Al-induced root growth inhibition is closely related with the Al accumulated in roots (Yu et al., 2015; Liu et al., 2018). For instance, Yu et al. (2015) proved that increasingly severe wheat root growth inhibition under Al stress conditions is accompanied by the accumulation of Al in root tips. Additionally, Liu et al. (2018) demonstrated that exacerbated root elogation inhibition under Al exposure mainly resulted from increased Al accumulation in root tips. Similar observations have also been reported for rice (Zhu et al., 2017) and Arabidopsis (Lou et al., 2019). Consistent with the greater inhibition of root growth in the Al-sensitive genotype YX02, the Al content was higher in YX02 than in the Al-resistant genotype YX01 (Figure 2D). Hence, the results of the current and previous studies suggest that in herbaceous plants as well as in coniferous tree species, such as Chinese fir, relative root elogation may be useful for distinguishing among genotypes with varying sensitivities to Al stress. Moreover, our results further confirmed previous findings and imply that Al-induced root growth inhibition is related to increased Al accumulation in root tips (Liu et al., 2018). The ROS-induced oxidative damage is a typical consequence of an exposure to excessive amounts of metals, including Al (Xu et al., 2012; Jin et al., 2013; Sun et al., 2015; Liu et al., 2018; Chen et al., 2020). Thus, oxidative damage is often used as a criterion for the occurrence of Al toxicity. Therefore, the H_2_O_2_ content (i.e., a typical ROS) and oxidative damage were analyzed in Chinese fir. In this study, the higher MDA content resulting from the greater accumulation of H_2_O_2_ in the YX02 roots than in the YX01 roots indicated the oxidative damage was more severe in of the Al-sensitive genotype than in the Al-resistant genotype (Figures 2A,B). It has been reported that antioxidant enzyme is vital for regulating oxidative damage in plants under Al stress (Xu et al., 2012). Increasing evidence showed that differences in response to oxidative damage responsible for plant difference in Al tolerance (Sun et al., 2014, 2015). For example, Yu et al. (2019) showed that enhancing antioxidant enzyme activities ameliorated Al resistance in both wheat cultivars, and a more prominent increased were observed in Al-tolerant one. Similar results also found in maize (Giannakoula et al., 2010). In this study, the severe oxidative damage observed in YX02 is associate with the significantly lower CAT, SOD, POD, and APX activities in YX02 than in YX01 under Al stress conditions (Figure 3), further confirmed previous results. Hence, above results imply that the more serious Al-induced oxidative damage observed in YX02 than in YX01 is due to a lower ROS scavenging capacity, which consequently increases H_2_O_2_ levels, thus leading to serous oxidative stress that restricts root growth (Liu et al., 2018).

In most cases, the Al accumulated in Al-stressed roots is associated with the binding of Al by the cell wall, which is the major Al-binding site in roots. Clarkson (1967) reported that 85%–90% of the Al accumulating in roots of barley (*Hordeum vulgare* L.) is located in the cell wall. Similarly, Liu et al. (2019) found that the Al accumulated in the cell walls of rice and wheat plants with different genotypes represents 64.9%–91.6% of the total Al content in the roots. Binding of Al to cell wall may destroy its structure, thus reduce cell wall extensibility. Indeed, previous study demonstrated that disorganized distribution of homogalacturonan (major component of cell wall pectin) epitopes is responsible for Al-induced root elongation inhibition (Li et al., 2009). Therefore, the cell wall is critical for regulating Al tolerance. Recent evidence suggests that pectin and HC are the two major fractions of cell wall responsible for binding of metal ions, including Al, iron and cadmium (Yang et al., 2011b; Zhu et al., 2014; Xu et al., 2015; Sun et al., 2016). In the current study, we found that pectin and HC1 make a great contribution to Al accumulation in cell wall as indicated by large portion of Al accumulated cell wall was found in these two components (Figure 4). Furthermore, the Al levels in the cell wall, pectin, HC1, and HC2 were greater in the roots of the Al-sensitive genotype YX02 than in the roots of the Al-resistant genotype YX01 (Figure 4). Accordingly, the Al accumulated in the pectin and HC fractions contribute greatly to the difference in the Al tolerance between Chinese fir genotypes. Generally, the binding capacity of cell wall for metal ions is related to the abundance of pectin and HC. For example, Eticha et al. (2005) reported that an Al treatment of maize (*Zea mays* L.) results in an increase in the root Al content of all cultivars, with a more prominent increased of Al were found in Al-sensitive cultivars due to higher accumulation of pectin in the roots. Similar results also reported for buckwheat, the results showed that the more significant accumulation of Al in roots of Al-sensitive cultivar “Liuku2” is associated with the higher increase and proportion of low-methylated pectin in its roots (Yang et al., 2011a). Additionally, Zhu et al. (2012b) proved that in the Arabidopsis mutant *xth31*, defects in xyloglucan synthesis substantially enhance plant Al-resistance because of a decrease in the cell wall HC content, which inhibits the ability of the roots to retain Al. Consistent with these earlier findings, we observed that an exposure to Al stress significantly increased the pectin and HC1 contents of both examined Chinese fir genotypes, although the increase was considerably more in the Al-sensitive genotype YX02 (Figure 5). This observation was in accordance with the increased Al content in the pectin and HC of YX02 (Figure 4). Therefore, these results are in agreement with previous findings, and imply that Al-induced increases in pectin and HC contents contribute greatly to the accumulation of Al in the cell wall and its genotypic difference in Al tolerance. In addition to the pectin and HC contents, the DM is another factor influencing the capacity of the cell wall to bind Al. Specifically, a lower DM can increase the number of carboxylic groups, which are potential serve as Al-binding sites. An earlier investigation revealed that the higher retention of Al in the cell wall of Al-sensitive rice cultivar is because of an increase in PME activity and a decrease in the DM, ultimately leading to enhanced plant sensitive to Al (Yang et al., 2008). Sun et al. (2016) also showed that in Al-sensitive wheat plants, eliminating the Al-induced accumulation of endogenous nitric oxide (NO) generation by a NO scavenger greatly decreased Al accumulation in cell wall via decreased PME activity and increased DM. Other studies have provided evidence of a negative correlation between the cell wall Al content and the DM (Eticha et al., 2005; Zhang et al., 2011; Yu et al., 2015; Sun et al., 2016). Therefore, the greater retention of Al in the YX02 cell wall than in the YX01 cell wall is likely associated with a decrease in the cell wall DM under Al stress conditions (Figure 6A). Pectin demethylation is reportedly regulated by PME activity (Xu et al., 2015). Consistent with decrease in the DM in both analyzed Chinese fir genotypes under Al stress, the Al treatment significantly increased the PME activity, especially in YX02 (Figure 6B). Considered together, these results suggest that the greater retention of Al in the cell wall of the Al-sensitive genotype YX02 is likely because of an increase in the pectin and HC contents, an enhancement of PME activity, and a decrease in the DM of the cell wall. Several reports have confirmed the metal ion-binding capacity of cell walls can be altered by modifying the cell wall under abiotic stress conditions. For instance, the Cd adsorption capacity of Arabidopsis plants increases in a phosphorus-deficient environment because of a decrease in the pectin and HC1 contents. Similarly, Ye et al. (2015) found that in tomato (*Solanum lycopersicum*), the application of exogenous NO enhances the ability of the cell wall to adsorb Fe by decreasing the cell wall DM via increased PME activity. In an earlier study, we proved that the Fe-binding capacity of the cell wall under Cd stress conditions increases as the pectin and HC contents increase and the DM decreases (Xu et al., 2015). In a recent study, Yang et al. (2019) observed that manganese (Mn) stress enhances the Mn-binding capacity of the cell wall by increasing the pectin content and PME and decreasing the DM in Mn-sensitive genotype of sugarcane (*Saccharum officinarum* L.). These results further support the importance of the regulatory effects of cell wall modifications on the capacity to bind metal ions. In line with these findings, we observed that an exposure to Al stress increasing the pectin and HC contents and the PME activity, while decreasing the DM in YX02, all of which enhance the ability of the cell wall to bind Al. These changes help to explain the greater adsorption of Al by the YX02 cell wall than by the YX01 cell wall (Figure 7).

In conclusion, the results of this study indicate that the differences in the Al tolerance of Chinese fir genotypes are associated with variations in Al-induced cell wall modifications and antioxidant enzyme activities. In response to toxic Al levels, the Al-sensitive genotype YX02 significantly increases its pectin and HC contents along with its PME activity, thereby substantially decreasing the cell wall DM, which enhances the ability of the cell wall to retain Al. Consequently, the Al-induced roots growth inhibition is exacerbated. Furthermore, the limited Al-induced stimulation of antioxidant enzymes in YX02 increases the ROS accumulation, resulting in severe oxidative stress.

## Data Availability Statement

The original contributions presented in the study are included in the article/supplementary material, further inquiries can be directed to the corresponding authors.

## Author Contributions

SX: data collection, methodology, analysis, and writing. LW, LL, MZ, and YT: data collection and analysis. GC and KL: writing original draft. YY: conceptualization, writing original draft, analysis, methodology, and funding acquisition. All authors contributed to the article and approved the submitted version.

## Conflict of Interest

The authors declare that the research was conducted in the absence of any commercial or financial relationships that could be construed as a potential conflict of interest.

## Publisher’s Note

All claims expressed in this article are solely those of the authors and do not necessarily represent those of their affiliated organizations, or those of the publisher, the editors and the reviewers. Any product that may be evaluated in this article, or claim that may be made by its manufacturer, is not guaranteed or endorsed by the publisher.
